# Explainable AI Method for Tinnitus Diagnosis via Neighbor-Augmented Knowledge Graph and Traditional Chinese Medicine: Development and Validation Study

**DOI:** 10.2196/57678

**Published:** 2024-06-10

**Authors:** Ziming Yin, Zhongling Kuang, Haopeng Zhang, Yu Guo, Ting Li, Zhengkun Wu, Lihua Wang

**Affiliations:** 1 School of Health Science and Engineering University of Shanghai for Science and Technology Shanghai China; 2 Department of Otolaryngology Shanghai Municipal Hospital of Traditional Chinese Medicine Shanghai University of Traditional Chinese Medicine Shanghai China

**Keywords:** knowledge graph, syndrome differentiation, tinnitus, traditional Chinese medicine, explainable, ear, audiology, TCM, algorithm, diagnosis, AI, artificial intelligence

## Abstract

**Background:**

Tinnitus diagnosis poses a challenge in otolaryngology owing to an extremely complex pathogenesis, lack of effective objectification methods, and factor-affected diagnosis. There is currently a lack of explainable auxiliary diagnostic tools for tinnitus in clinical practice.

**Objective:**

This study aims to develop a diagnostic model using an explainable artificial intelligence (AI) method to address the issue of low accuracy in tinnitus diagnosis.

**Methods:**

In this study, a knowledge graph–based tinnitus diagnostic method was developed by combining clinical medical knowledge with electronic medical records. Electronic medical record data from 1267 patients were integrated with traditional Chinese clinical medical knowledge to construct a tinnitus knowledge graph. Subsequently, weights were introduced, which measured patient similarity in the knowledge graph based on mutual information values. Finally, a collaborative neighbor algorithm was proposed, which scored patient similarity to obtain the recommended diagnosis. We conducted 2 group experiments and 1 case derivation to explore the effectiveness of our models and compared the models with state-of-the-art graph algorithms and other explainable machine learning models.

**Results:**

The experimental results indicate that the method achieved 99.4% accuracy, 98.5% sensitivity, 99.6% specificity, 98.7% precision, 98.6% *F*_1_-score, and 99% area under the receiver operating characteristic curve for the inference of 5 tinnitus subtypes among 253 test patients. Additionally, it demonstrated good interpretability. The topological structure of knowledge graphs provides transparency that can explain the reasons for the similarity between patients.

**Conclusions:**

This method provides doctors with a reliable and explainable diagnostic tool that is expected to improve tinnitus diagnosis accuracy.

## Introduction

Tinnitus is a common refractory disease in the field of otolaryngology, and its diagnosis has always been a cutting-edge research topic in audiology. With changes in the social environment and an accelerated pace of life, an increasing number of patients, particularly among the younger generation, have sought medical assistance for tinnitus as their primary complaint in the last decade. Globally, approximately 14% (95% CI 0.8%-1.6%) of adults are affected by tinnitus [1,2], which can cause stress, anxiety, and depression [3]. Distress and hearing impairment brought on by the disease can affect cognitive abilities and lead to suicidal tendencies in severe cases, greatly affecting the work and daily lives of patients [4].

The pathogenesis of tinnitus is extremely complex and not fully understood. Currently, no effective objectification methods are available. Traditional Chinese medicine (TCM) classifies tinnitus into 5 different syndrome patterns: wind fire attacking internally (WFAI), liver fire bearing upward (LFBU), phlegm fire stagnation internally (PFSI), Qi deficiency of the spleen and stomach (QDSS), and kidney essence deficiency (KED). The diagnosis of tinnitus remains a challenge in medical science because it is influenced by several complex factors [5,6], including individual differences among patients and atypical symptom presentations. Clinical diagnosis relies heavily on the personal knowledge and clinical experience of doctors, thereby introducing subjectivity, uncertainty, and ambiguity. Consequently, achieving a high tinnitus diagnostic accuracy becomes difficult. Therefore, tinnitus diagnosis remains an urgent issue requiring further exploration and resolution by medical researchers.

Previous studies have focused on the use of artificial intelligence (AI) to assist doctors in diagnosing tinnitus and improving diagnostic accuracy. Liu et al [7] proposed a meta-learning method based on lateral perception for cross–data set tinnitus diagnosis. Sun et al [8] used a support vector machine classifier to distinguish between patients with tinnitus and healthy individuals. Shoushtarian et al [9] used a naive Bayes algorithm to classify patients with tinnitus and control groups. Sanders et al [10] used a spiking neural network model to classify patients with tinnitus into 2 groups based on different classification criteria. Manta et al [11] used clinical data and patient features to build a machine learning (ML) model for classifying the degree of tinnitus-related distress in individuals and their ears. Allgaier et al [12] used a gradient-boosting engine to classify transient tinnitus. Rodrigo et al [13] used a decision tree model to identify variables related to the success of internet-based cognitive behavioral therapy for tinnitus. Liu et al [14] used a support vector machine model to explore cortical or subcortical morphological neuroimaging biomarkers that effectively distinguished patients with tinnitus from healthy individuals. Niemann et al [15] proposed a LASSO model to predict the severity of depression in patients with tinnitus. Although previous studies have achieved success using their respective data sets, the developed ML- or deep learning–based methods are entirely data-driven modeling approaches that do not make full use of existing medical knowledge. Models built using such methods are equivalent to “black boxes” for doctors, lack interpretability, and are not conducive to clinical promotion and application.

In this study, the aim is to incorporate clinical medical knowledge into a diagnostic model, enabling the integration of knowledge and data for interpretable results. Knowledge graph–based modeling methods offer solutions to such issues by using a novel knowledge representation format that connects entities and concepts in an objective world using semantic relationships. Such methods offer reasoning and interpretability that are highly sought after by both medical practitioners and academia. Li et al [16] used a knowledge graph to predict diabetic macular edema, overcoming the limitations of traditional ML and data-mining techniques that deal with missing feature values. Zhou et al [17] used 124 medical records to construct a knowledge graph for recommending hypertension medication. Lyu et al [18] created a knowledge graph for diabetic nephropathy diagnosis using patient data. Lin et al [19] extracted knowledge from medical texts and historical prescription data to construct a medical knowledge graph and accurately detect clinical prescription risks. Recently, knowledge graph applications have expanded to TCM; for instance, Yang et al [20] built a knowledge graph to extract medical information from TCM case records. Xie et al [21] constructed a knowledge graph using ancient Chinese medical books to infer symptoms and syndromes. Yang et al [22] used electronic medical records (EMRs) to build a knowledge graph, transforming TCM diagnostic issues into multilabel classification problems. Lan et al [23] integrated knowledge graphs with graph neural networks to introduce graph-based supervised contrastive learning, effectively enabling the classification of TCM texts. However, no previous studies have used knowledge graphs in the complex medical field of tinnitus diagnosis. Therefore, this study focuses on knowledge graph technology to assist doctors in tinnitus diagnosis and improve diagnostic accuracy.

This paper aims to establish a comprehensive knowledge graph in TCM specifically tailored for tinnitus. Leveraging this knowledge graph, we propose a novel method for calculating patient similarity. This method takes into account the weighting of symptom-syndrome type relationships, thereby facilitating the inference of syndrome types in patients with tinnitus according to TCM principles. By implementing this approach, clinicians can increase the accuracy of tinnitus diagnosis within the realm of TCM.

In general, we make several noteworthy contributions as follows:

- We propose a method for tinnitus knowledge graph construction based on heterogeneous patient EMRs and TCM clinical knowledge.
- We introduce weights to measure patient similarity into the tinnitus knowledge graph using a method based on prior probabilities and mutual information values.
- A collaborative neighbor algorithm that uses patient similarity scores to obtain recommended diagnostic results is proposed to assist doctors in understanding the model-generated conclusions, thereby improving the accuracy of tinnitus diagnosis.

## Methods

### Patients

For this study, we collected the EMRs of 1267 patients with tinnitus who visited the ear, nose, and throat departments of 11 medical institutions in Shanghai, China, from November 2019 to July 2023. The inclusion criteria included (1) tinnitus as the primary complaint and (2) the ability to communicate normally. The exclusion criteria included (1) objective tinnitus, (2) nonotogenic tinnitus caused by factors such as endocrine and blood disorders, (3) tinnitus caused by head or ear trauma, and (4) difficulties in communication or severe psychiatric history that could hinder follow-up compliance. After screening the data for quality, 1265 cases were included for further analysis.

**Figure 1:**
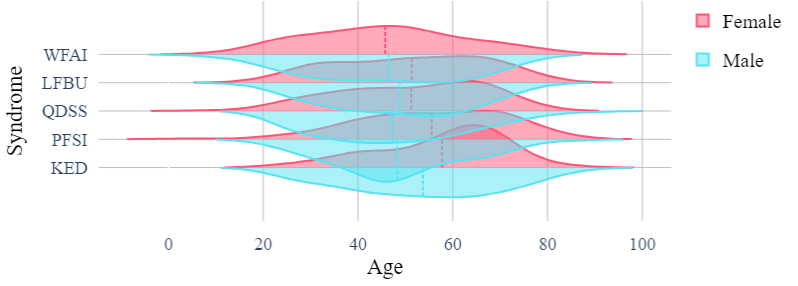
Age distribution of different syndromes by sex. KED: kidney essence deficiency; LFBU: liver fire bearing upward; PFSI: phlegm fire stagnation internally; QDSS: Qi deficiency of the spleen and stomach; WFAI: wind fire attacking internally.

**Figure 2:**
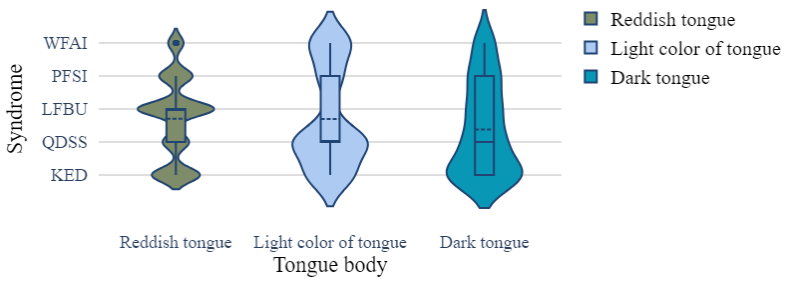
The tongue body distribution of different syndrome types. KED: kidney essence deficiency; LFBU: liver fire bearing upward; PFSI: phlegm fire stagnation internally; QDSS: Qi deficiency of the spleen and stomach; WFAI: wind fire attacking internally.

**Figure 3:**
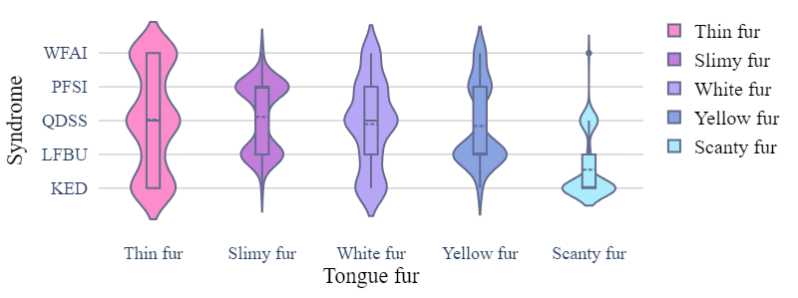
The tongue fur distribution of different syndrome types. KED: kidney essence deficiency; LFBU: liver fire bearing upward; PFSI: phlegm fire stagnation internally; QDSS: Qi deficiency of the spleen and stomach; WFAI: wind fire attacking internally.

**Figure 4:**
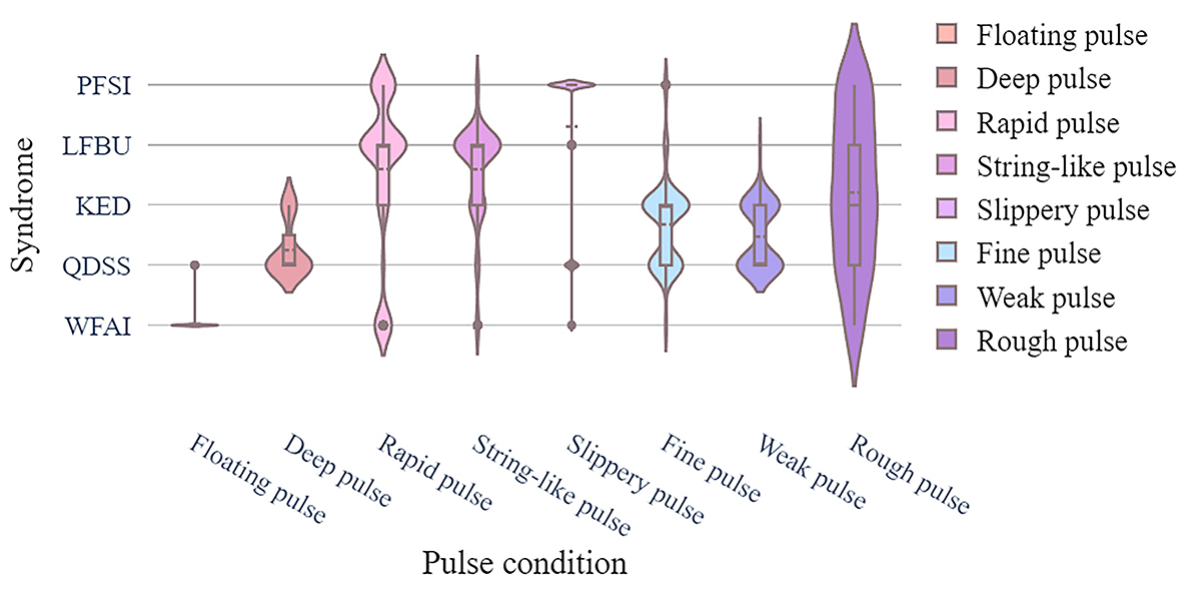
The pulse condition distribution of different syndrome types. KED: kidney essence deficiency; LFBU: liver fire bearing upward; PFSI: phlegm fire stagnation internally; QDSS: Qi deficiency of the spleen and stomach; WFAI: wind fire attacking internally.

The clinical EMR data set recorded medical data of real patients including the relationship between patient symptoms and disease, which was crucial for disease diagnosis. The data set contained patient information such as age, sex, inducement, medical history, tinnitus sound, accompanying symptoms, tongue coating, pulse condition, TCM syndrome differentiation, and sleep status. Each patient had a clear diagnosis that could be classified into 1 of 5 categories: WFAI, LFBU, PFSI, QDSS, and KED. Statistical data are presented in Figures 1-4 .

### Ethical Considerations

This study’s protocol was approved by the ethics committee of the Shanghai Municipal Hospital of Traditional Chinese Medicine, Shanghai, China (2021SHL-KY-70).

The data was anonymized in order to protect patient privacy. Patients could receive free examinations and treatments throughout the entire process, so no compensation was provided.

### Clinical Decision Support for Tinnitus

#### Overview

**Figure 5:**
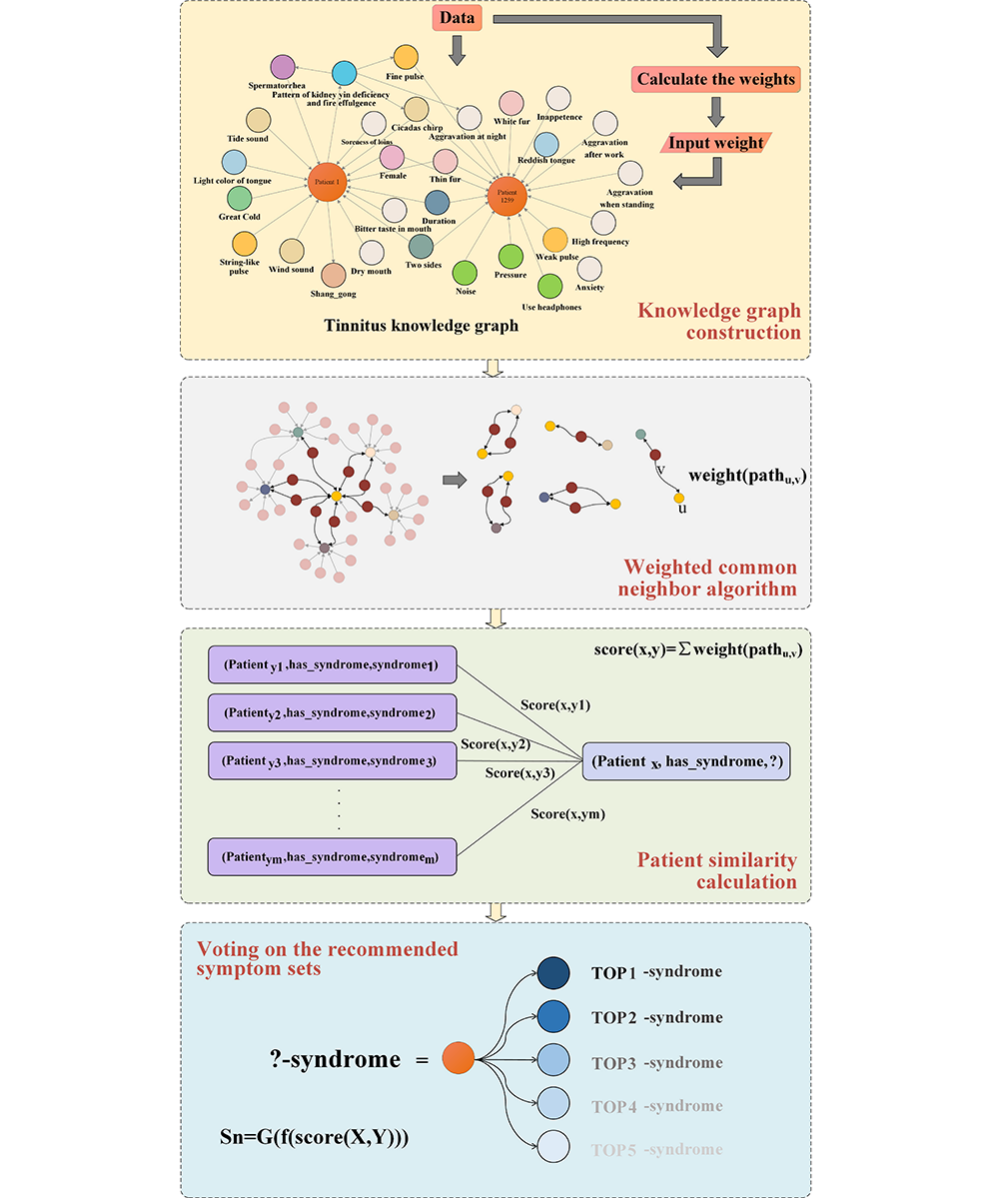
Overall framework of the proposed method.

To integrate patient EMRs with diagnostic knowledge from TCM textbooks, we constructed a knowledge graph using a combined “top-down” and “bottom-up” approach [24]. First, a patient-centered knowledge graph was developed using EMRs. Then, the knowledge graph was enriched with tinnitus diagnostic knowledge from TCM textbooks. Finally, we used a mutual information–based weight calculation method to enhance the knowledge graph by fusing patient case data with diagnostic knowledge. The resulting knowledge graph simulated the diagnostic reasoning processes of experienced physicians. The entire method consisted of three steps: (1) building a weighted tinnitus knowledge graph, (2) finding and scoring common neighbors, and (3) predicting syndrome patterns based on patient similarity. The overall framework is illustrated in Figure 5.

#### Knowledge Graph of Tinnitus Based on Heterogeneous Sources

In response to the diagnostic needs of tinnitus in TCM, the ontology structure of a tinnitus medical knowledge graph should revolve around symptoms, syndrome patterns, diseases, drugs, and treatment methods. For this study, we extracted such common concepts from expert-reviewed EMRs and classic medical textbooks, constructed a conceptual knowledge system, and built a top-level ontology structure. Natural language processing techniques [25] were used to extract entities and relationships from the patient EMRs based on a defined conceptual knowledge system for tinnitus. By applying certain rules and conducting string matching within the text, we extracted 15 and 10 categories of entities and relationships from the 1265 EMR records, respectively. Once the entity types and hierarchy were determined, we embedded the data into the conceptual knowledge system and established a patient-centric tinnitus knowledge graph in the form of a triple, which maximized the retention of both explicit and implicit diagnostic information.

Furthermore, we enhanced the constructed tinnitus knowledge graph using knowledge extracted from authoritative medical textbooks to supplement tinnitus knowledge information that was not fully expressed in EMRs. Together with the EMR knowledge graph, a complete tinnitus knowledge graph was developed. The knowledge we selected came from 2 classic Chinese medicine textbooks [26,27], from which we extracted basic concepts related to tinnitus including TCM syndromes, prescriptions, Chinese medicinal herbs, and treatment methods to construct the TCM knowledge graph.

#### Heterogeneous Knowledge Fusion

Redundancy in the entities and relationships extracted from heterogeneous sources was observed owing to the different sources of data and knowledge. Therefore, knowledge fusion was required. First, data normalization and entity alignment were performed to standardize the named entities extracted from multiple data sources. The entities were associated using string-matching and similarity-calculation methods. As entity and attribute texts were relatively short, a lower similarity threshold was more appropriate; therefore, the similarity judgment threshold was set as 0.6 to prevent errors and omissions. The entity similarity calculation results are listed in Table 1. As the knowledge graph was established in Chinese, we calculated the similarity of the Chinese strings.

**Figure 6:**
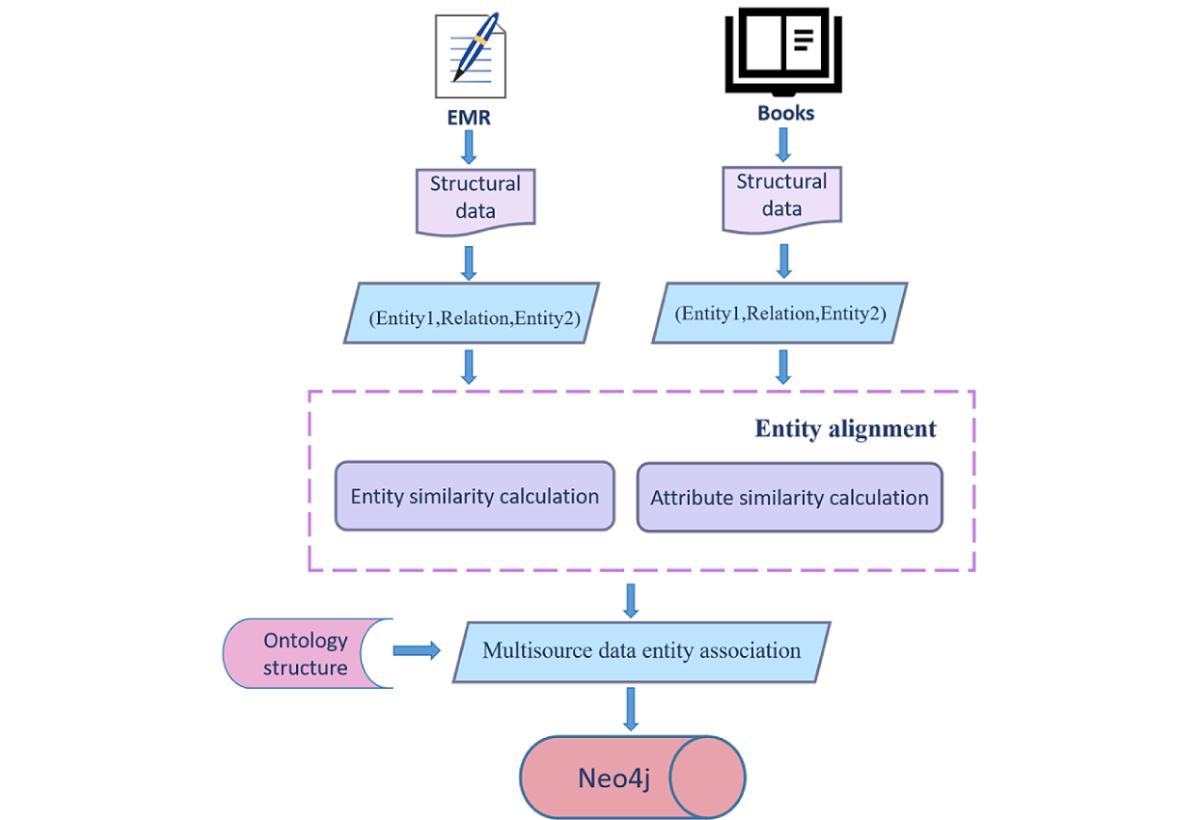
Tinnitus knowledge graph fusion flowchart. EMR: electronic medical record.

Then, a matching path was built from the tinnitus ontology–based knowledge graph entity to the EMR-based knowledge graph entity. Patient data were linked to diagnostic knowledge through an ontology. The 2 knowledge graphs were linked by unifying entities with duplicate meanings in the 2 graphs. Manual verification was performed to ensure the accuracy of the knowledge graph. The specific method is illustrated in Figure 6. Finally, the tinnitus knowledge graph consisted of 1247 entities and 9234 relationships.

**Table 1:** Entity similarity calculation results.

Standardized and ambiguous entities (Chinese)		Similarity
**WFAI^a^**		
	风热外侵证 (wind-heat invasion syndrome)	0.8
	风热外犯证 (wind-heat exterior syndrome)	0.6
	风热外侵证 (wind-heat exterior assault syndrome)	0.8
**LFBU^b^**		
	肝火上炎证 (liver fire flaming upward syndrome)	0.8
	肝热上扰证 (liver heat disturbing upward syndrome)	0.8
	肝火上扰清窍证 (liver fire disturbing upward and disturbing clearing orifices syndrome)	0.83
**QDSS^c^**		
	脾胃虚证 (spleen and stomach deficiency syndrome)	0.89
	脾胃虚弱证 (spleen and stomach weakness syndrome)	0.8
**PFSI^d^**		
	痰火壅结证 (phlegm-fire concretions syndrome)	0.8
**KED^e^**		
	肾精不足证 (kidney essence insufficiency syndrome)	0.6
	肾精亏虚证 (kidney essence deficiency syndrome)	0.8
	肾虚精亏证 (kidney deficiency and essence deficiency syndrome)	0.99
	肾精亏耗证 (kidney essence consumption syndrome)	0.8

^a^WFAI: wind fire attacking internally.

^b^LFBU: liver fire bearing upward.

^c^QDSS: Qi deficiency of the spleen and stomach.

^d^PFSI: phlegm fire stagnation internally.

^e^KED: kidney essence deficiency.

#### Calculation of Knowledge Graph Relationship Weights Based on Mutual Information

Considering the varying importance of different entities for different syndrome patterns, the imbalance in data categories, and the varying amount of information carried by symptoms, the calculation of weights required consideration of entities’ importance for diagnostic pattern identification and information content carried by the entities themselves. The data used for weight calculation were derived from real clinical case data used for constructing the knowledge graph. First, the mutual information value (*w*_if_) possessed by each entity was obtained using the mutual information method. The obtained value represented the extent to which a variable could acquire diagnostic pattern information.

For a given set of entities *X* = {*x*_1_, *x*_2_, ..., *x*_n_} with corresponding probabilities *P* = {*p*_1_, *p*_2_, ..., *p*_n_}, the target variable to be measured was the diagnostic pattern *Y*. By calculating the overall entropy *H*(), conditional entropy *H*(*Y*|*X*), and mutual information value *Gain*(*S*,*x*), the degree to which the diagnostic pattern was determined based on the entity values or the weight value *w*_if_ of the entity was calculated. The calculations were performed using equations 1-3.


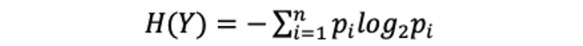
 (1)



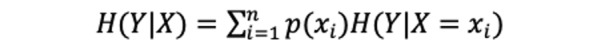
 (2)

*w_if_* = *Gain*(*Y*,*X*) = *H*(*Y*) – *H*(*Y*|*X*) (3)

Further, the feature weights were calculated based on the syndrome patterns under the prior conditions. The probability of each symptom appearing under different syndrome patterns was obtained using statistical methods such as:

*w_sd_* = *p*(*sym_i_*|*sd_j_*) (4)

where *sym* = {*sym_1_, sym_2_, ..., sym_n_*} represents the symptom set and *sd* = {*sd_1_, sd_2_, ..., sd_m_*} represents the diagnostic pattern set. Finally, the edge weight from node *u* to node *v* was defined using equation 5.

*Weight*(*u*,*v*) = *w_if_* + *w_sd_* (5)

The weights of various symptoms under different syndrome patterns are presented in Table 2.

**Table 2:** Partial weight value of symptom-syndrome type.

Symptom		Weight
**KED^a^**		
	Spermatorrhea	1.435
	Soreness of loins	1.4213
	Dreaminess	1.4104
	Wake up early in the morning	1.3868
	Deficiency and insomnia	1.3856
	Aggravation at night	1.167
	Cicadas chirp	1.1559
	Fine pulse	1.1448
	Scanty fur	0.7142
	Duration	0.6991
**LFBU^b^**		
	Irritable	1.2376
	Restlessness and insomnia	1.1196
	Wind sound	1.0271
	String-like pulse	1.0056
	Tide sound	1.0030
	Yellow fur	0.9118
	Reddish tongue	0.8992
	Duration	0.7036
	Dry mouth	0.6855
	Bitter taste in mouth	0.6558
**PFSI^c^**		
	Tastelessness	1.1953
	Dizziness and heaviness	1.1488
	Aural fullness	1.1216
	Ear distension	1.0899
	Slippery pulse	0.9121
	Slimy fur	0.8342
	Duration	0.7113
	Yellow fur	0.6895
	Hearing loss	0.6495
	Reddish tongue	0.6440
**WFAI^d^**		
	Cold or rhinitis	1.2089
	Tinnitus onset within a month	1.1398
	Low voice	1.1398
	Thin fur	1.0286
	Floating pulse	0.9563
	Duration	0.6903
	Light color of tongue	0.6664
	Yellow fur	0.5082
	Hearing loss	0.5032
	Dreaminess	0.4993
**QDSS^e^**		
	Feeling emptiness in ear	1.2615
	Aggravation after work	1.1813
	Aggravation when standing up	1.1562
	Fine pulse	1.0782
	Duration	0.7370
	Thin fur	0.7022
	Light color of tongue	0.6745
	Anxiety	0.6596
	Hearing loss	0.6444
	Dreaminess	0.4865

^a^KED: kidney essence deficiency.

^b^LFBU: liver fire bearing upward.

^c^PFSI: phlegm fire stagnation internally.

^d^WFAI: wind fire attacking internally.

^e^ODSS: Qi deficiency of the spleen and stomach.

#### Patient Similarity Scoring Based on Weighted Common Neighbor Algorithm

**Figure 7:**
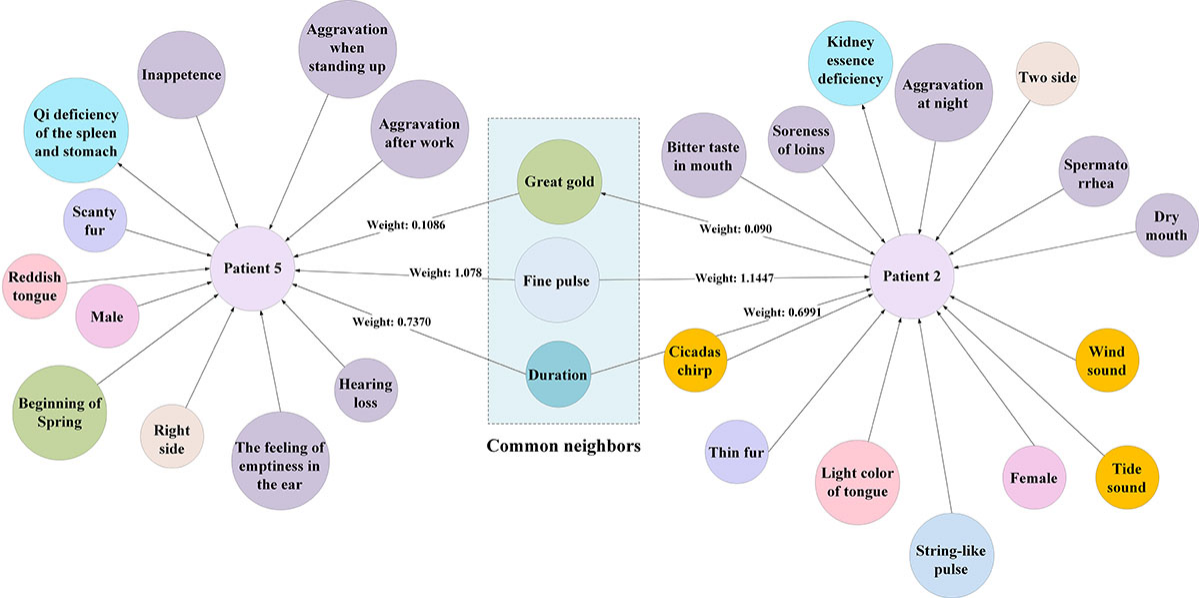
Sketch map of common neighbors between different syndromes.

**Figure 8:**
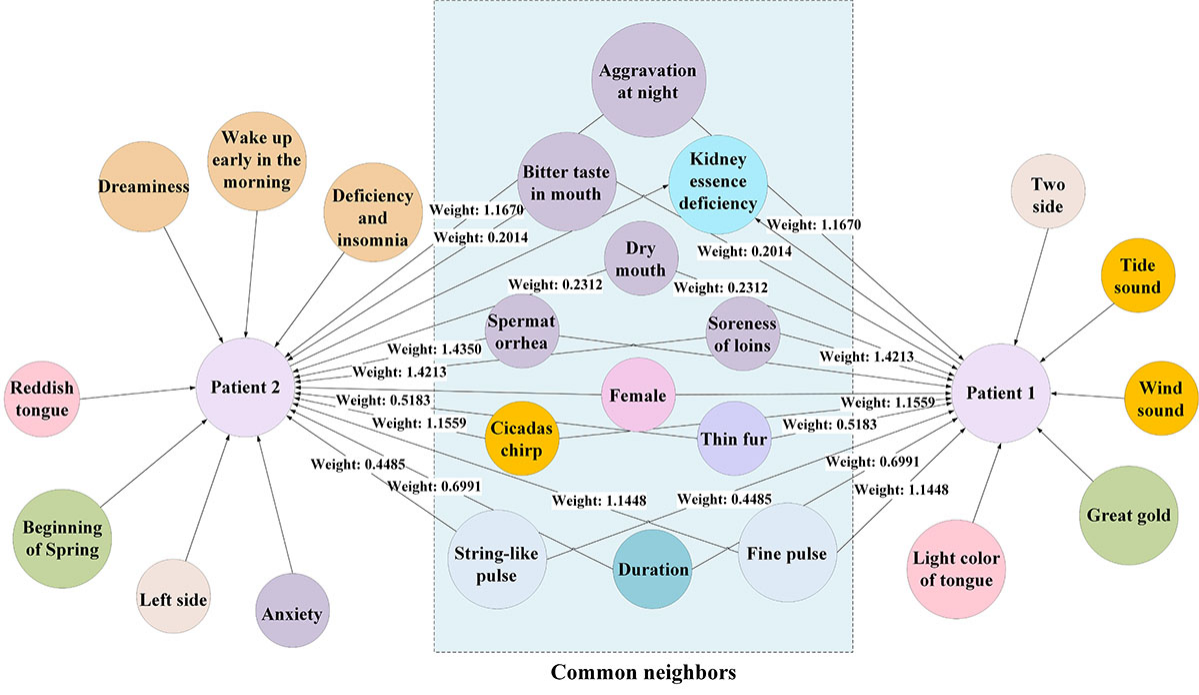
Sketch map of common neighbors between same syndromes.

By transforming the TCM syndrome diagnostic problem into a prediction problem of linked patient nodes to TCM syndrome nodes, the similarity between 2 patients was calculated to obtain TCM syndrome similarity. For 2 patients, the higher the similarity, the greater the likelihood of having the same diagnostic result. This study measured the similarity using common features. In the knowledge graph, the higher the number of common neighbors to 2 patient nodes, the greater the likelihood of them belonging to the same community (linked to the same TCM syndrome node). The common neighbor graph of patients with different TCM syndromes is shown in Figure 7, where fewer common neighbors were observed. The common neighbor graph of patient 1 and patient 2 with the same TCM syndrome is shown in Figure 8, where more common neighbors were observed; however, different nodes had different importance. In TCM, the importance of pulse condition is greater than that of tinnitus duration while diagnosing tinnitus. The edge weight values of continuous tinnitus and thin pulse-to-kidney deficiency syndrome were 0.6991 and 1.1448, respectively, as shown in Figure 7; however, even for the same pulse condition, the importance varied for different TCM syndromes. In Figure 8, the edge weight values of thin pulse to QDSS and KED syndromes were 1.078 and 1.1447, respectively. Therefore, considering the edge weights of common neighbors to the patient nodes and calculating the score of common neighbors based on the edge weight values were essential when counting the number of common neighbors between patient nodes.

The similarity scoring function between patients *x* and *y* was defined by equation 6.


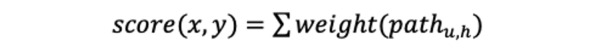
 (6)


where *X* = {*u_1_, u_2_, ..., u_m_*} and *Y* = {*v_1_, v_2_, ..., v_n_*} represent the sets of neighboring nodes for patients *x* and *y*, respectively; *Path_u,h,v_* = (*u, h, v*) denotes the 2-hop path from node *u* to node *v*, where *h* represents the common neighbor of nodes *u* and *v*; *Path_u,h_* = (*u, h*) represents the path from node *u* to the common neighbor *h*; and *weight*(*path_u,h_*) indicates the weight of the path.

When 2 paths with a hop count of 2 between the patient nodes existed, the weights of the paths were calculated to obtain a similarity score list for the patients. The list was then sorted in descending order, and the top 20 patient node syndromes with the highest scores were counted, which represented the most frequently occurring syndrome. Finally, the recommended syndrome was obtained.

*S_n_* = *G*(*f*_20_(score(*X*,*Y*))) (7)

where *G* denotes a frequency-counting method in which *X* and *Y* represent sets of patient nodes. *f*_20_() was used to obtain the top 20 patient syndromes based on the scores.

### Experimental Design

In total, 2 experiments were conducted to verify the effectiveness of the proposed method. The first experiment was performed to compare the proposed method with similar graph algorithms, while the second experiment was performed to compare the proposed method with other common explainable ML methods. The evaluation metrics of the algorithm are accuracy, precision, sensitivity, specificity, *F*_1_-score, area under receiver operating characteristic curve (AUC), etc. To demonstrate the interpretability of our method, we selected a tinnitus case for result interpretation to showcase the inference process and interpretability of our method.

## Results

### Performance Verification

For a given knowledge graph, we extracted the patient nodes and their neighboring nodes to form a knowledge network. The node and edge sets in the knowledge network were divided into training and testing sets. The testing set did not contain syndrome entities. To reasonably divide the training and testing sets, we used a stratified sampling cross-validation method of randomly dividing the network node and edge sets into 5 subsets: 1 subset as the testing set, and the other 4 subsets as the training set. The training set served as a known network, whereas the testing set was used to verify the syndrome prediction results and evaluate the accuracy of the syndrome prediction algorithm.

### Evaluation Outcomes

#### Comparison With Similar Graph Algorithms

The proposed method was compared with similar graph algorithms such as CommonNeighbors and Adamic-Adar. CommonNeighbors is a common graph algorithm used to infer the potential relationships and proximity between 2 nodes [28]; however, the differences between common neighbors are not considered. Adamic-Adar is a typical algorithm for determining the closeness of 2 points by measuring the outdegree of common neighbors [29]. ResourceAllocation calculates the closeness between 2 nodes using a set of neighboring nodes near the target node [30]. We added common neighbor edge weights based on CommonNeighbors. Unlike Adamic-Adar and ResourceAllocation, our weight calculation method considered each syndrome, which had a higher adaptability to TCM diagnosis by the doctors. The experimental results are listed in Table 3; our method outperformed similar graph algorithms in diagnosing each syndrome.

**Table 3:** Experimental results of graph algorithm comparison.

Evaluation indicators and models		KED^a^ (n=339)	LFBU^b^ (n=307)	PFSI^c^ (n=194)	QDSS^d^ (n=270)	WFAI^e^ (n=155)	Value, mean (SD)
**Average accuracy**							
	Common neighbors	0.978	0.978	0.982	0.983	0.988	0.982 (0.004)
	Adamic-Adar	0.979	0.979	0.978	0.983	0.989	0.982 (0.004)
	Resource allocation	0.918	0.944	0.961	0.936	0.974	0.947 (0.019)
	WeightedCommonNeighbors	0.990	0.994	0.995	0.992	0.998	0.994 (0.003)
**Average precision**							
	Common neighbors	0.939	0.941	0.952	0.982	0.971	0.957 (0.017)
	Adamic-Adar	0.940	0.949	0.932	0.981	0.971	0.955 (0.019)
	Resource allocation	0.794	0.893	0.930	0.860	0.948	0.885 (0.055)
	WeightedCommonNeighbors	0.970	0.987	0.993	0.986	1.000	0.987 (0.010)
**Average sensitivity**							
	Common neighbors	0.981	0.971	0.922	0.943	0.929	0.949 (0.023)
	Adamic-Adar	0.984	0.965	0.917	0.942	0.935	0.949 (0.023)
	Resource allocation	0.933	0.877	0.801	0.840	0.837	0.857 (0.045)
	WeightedCommonNeighbors	0.990	0.990	0.976	0.979	0.987	0.985 (0.006)
**Average *F*_1_-score**							
	Common neighbors	0.959	0.956	0.936	0.961	0.949	0.952 (0.009)
	Adamic-Adar	0.961	0.957	0.924	0.961	0.952	0.951 (0.014)
	Resource allocation	0.856	0.884	0.859	0.849	0.885	0.866 (0.015)
	WeightedCommonNeighbors	0.980	0.989	0.984	0.982	0.994	0.986 (0.005)
**Average specificity**							
	Common neighbors	0.978	0.980	0.993	0.995	0.996	0.988 (0.008)
	Adamic-Adar	0.978	0.983	0.989	0.995	0.996	0.988 (0.007)
	Resource allocation	0.914	0.966	0.990	0.963	0.994	0.965 (0.029)
	WeightedCommonNeighbors	0.989	0.996	0.999	0.996	1.000	0.996 (0.004)
**Average AUC^f^**							
	Common neighbors	0.979	0.976	0.958	0.969	0.963	0.969 (0.008)
	Adamic-Adar	0.981	0.974	0.953	0.969	0.966	0.968 (0.009)
	Resource allocation	0.923	0.922	0.895	0.901	0.915	0.911 (0.011)
	WeightedCommonNeighbors	0.990	0.993	0.987	0.988	0.994	0.990 (0.003)

^a^KED: kidney essence deficiency.

^b^LFBU: liver fire bearing upward.

^c^PFSI: phlegm fire stagnation internally.

^d^QDSS: Qi deficiency of the spleen and stomach.

^e^WFAI: wind fire attacking internally.

^f^AUC: area under receiver operating characteristic curve.

#### Comparison With Other Interpretable ML Methods

The proposed method was compared with common ML classification algorithms including decision tree, random forest, naive Bayes, logistic regression, and k-nearest neighbors algorithms. The results are presented in Table 4. The graph algorithm based on WightedCommonNeighbor outperformed other models in the comprehensive diagnosis of each syndrome on the same data set but was lower than the random forest model in terms of the AUC metric. Although the random forest model had a certain degree of interpretability, the overall complexity of model interpretation increased when a large number of decision trees were included. The higher the number of decision trees in the random forest model, the greater the difficulty of interpreting the relationships and decision processes within the model. Compared to the random forest model, our proposed method had higher interpretability and was more readily accepted by doctors.

**Table 4:** Experimental results of machine learning classification algorithm comparison.

Evaluation indicators and models		KED^a^	LFBU^b^	PFSI^c^	QDSS^d^	WFAI^e^	Value, mean (SD)
**Average accuracy**							
	WeightedCommonNeighbors	0.990	0.994	0.995	0.992	0.998	0.994 (0.003)
	Decision tree	0.975	0.975	0.978	0.970	0.984	0.976 (0.005)
	Random forest	0.987	0.982	0.985	0.987	0.994	0.987 (0.004)
	Naive Bayes	0.979	0.976	0.979	0.981	0.991	0.981 (0.005)
	Logistic regression	0.986	0.983	0.983	0.984	0.994	0.986 (0.004)
	KNN^f^	0.986	0.980	0.982	0.986	0.994	0.985 (0.005)
**Average precision**							
	WeightedCommonNeighbors	0.970	0.987	0.993	0.986	1.000	0.987 (0.010)
	Decision tree	0.950	0.951	0.917	0.943	0.937	0.939 (0.012)
	Random forest	0.974	0.950	0.970	0.982	0.963	0.968 (0.011)
	Naive Bayes	0.971	0.923	0.953	0.956	0.980	0.957 (0.019)
	Logistic regression	0.971	0.961	0.950	0.964	0.981	0.965 (0.010)
	KNN	0.974	0.938	0.958	0.978	0.980	0.966 (0.016)
**Average sensitivity**							
	WeightedCommonNeighbors	0.990	0.990	0.976	0.979	0.987	0.985 (0.006)
	Decision tree	0.959	0.945	0.943	0.915	0.936	0.939 (0.014)
	Random forest	0.976	0.977	0.933	0.956	0.987	0.966 (0.019)
	Naive Bayes	0.953	0.981	0.912	0.956	0.948	0.950 (0.022)
	Logistic regression	0.976	0.967	0.938	0.963	0.968	0.963 (0.013)
	KNN	0.973	0.984	0.923	0.956	0.968	0.961 (0.021)
**Average *F*_1_-score**							
	WeightedCommonNeighbors	0.980	0.989	0.984	0.982	0.994	0.986 (0.005)
	Decision tree	0.953	0.948	0.929	0.928	0.936	0.939 (0.010)
	Random forest	0.975	0.963	0.950	0.968	0.975	0.966 (0.009)
	Naive Bayes	0.961	0.951	0.932	0.955	0.964	0.953 (0.011)
	Logistic regression	0.974	0.964	0.943	0.963	0.974	0.964 (0.011)
	KNN	0.973	0.960	0.940	0.966	0.974	0.963 (0.012)
**Average specificity**							
	WeightedCommonNeighbors	0.989	0.996	0.999	0.996	1.000	0.996 (0.004)
	Decision tree	0.981	0.984	0.984	0.985	0.991	0.985 (0.003)
	Random forest	0.990	0.983	0.994	0.995	0.995	0.992 (0.005)
	Naive Bayes	0.989	0.974	0.992	0.988	0.997	0.988 (0.008)
	Logistic regression	0.989	0.988	0.991	0.990	0.997	0.991 (0.003)
	KNN	0.990	0.979	0.993	0.994	0.997	0.991 (0.006)
**Average AUC^g^**							
	WeightedCommonNeighbors	0.990	0.993	0.987	0.988	0.994	0.990 (0.003)
	Decision tree	0.970	0.964	0.964	0.950	0.963	0.962 (0.007)
	Random forest	0.995	0.998	0.996	0.997	1.000	0.997 (0.002)
	Naive Bayes	0.996	0.996	0.993	0.995	0.997	0.995 (0.001)
	Logistic regression	0.997	0.997	0.994	0.995	0.997	0.996 (0.001)
	KNN	0.993	0.993	0.977	0.988	0.993	0.989 (0.006)

^a^KED: kidney essence deficiency.

^b^LFBU: liver fire bearing upward.

^c^PFSI: phlegm fire stagnation internally.

^d^QDSS: Qi deficiency of the spleen and stomach.

^e^WFAI: wind fire attacking internally.

^f^KNN: k-nearest neighbor.

^g^AUC: area under receiver operating characteristic curve.

## Discussion

### Principal Findings

The experimental results show that the accuracy, sensitivity, specificity, precision, *F*_1_-score, and AUC of our proposed method all exceed 98% for 5 tinnitus subtypes. Compared to the traditional graph algorithm, our method comprehensively considers the number of neighboring nodes and the weight of edges for patient nodes. This method of calculating the strength of node connections and feature importance can more comprehensively measure the similarity between patient nodes. Further, by calculating the common neighbor score, the similarity between patient nodes can be quantitatively measured, providing a reliable quantitative indicator for the prediction problem of patient-to-syndrome node links. In addition, in the field of TCM, the impact of different features on diagnostic results may vary. This method considers the importance of features through edge weight values, making similarity calculations more realistic. By considering the edge weight values, the reasons for the formation of similarity between patient nodes and the importance of features can be explained, enhancing the interpretability of the model results. This method is not only applicable to the diagnosis of syndrome types in the field of TCM but can also be applied in other fields, especially in the similarity calculation problem that needs to consider feature importance and node correlation strength, which has universality.

**Figure 9:**
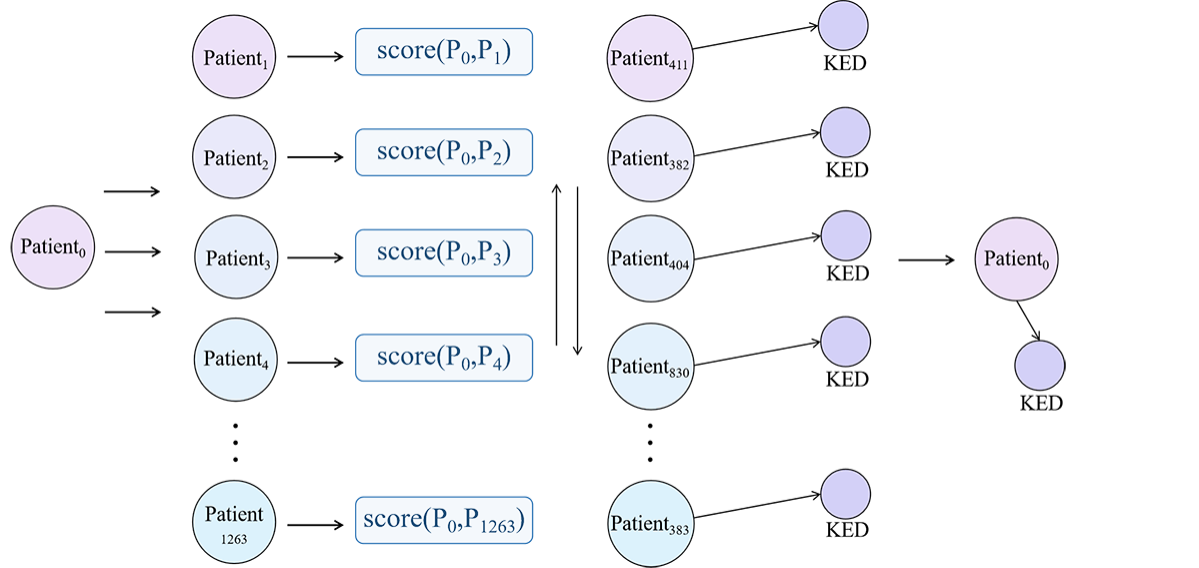
The inference process of patient syndrome patterns. KED: kidney essence deficiency.

In terms of interpretability, the proposed method integrated the knowledge of TCM differential diagnosis and clinical experience into a knowledge graph, which made the method more interpretable. To illustrate the explainability of our method, we randomly selected a patient from the patient records and used their medical information as input to the syndrome diagnosis algorithm, as shown in Figure 9. The patient information was input to the knowledge graph, where we searched for other patients who shared common neighbors with the selected patient. We calculated the common neighbor scores and returned the top k (k=20) patients with the highest scores. The results are summarized in Table 5. Based on the syndromes of the top k patients that were most similar to the target patient, we deduced that the predicted syndrome of the target patient was KED, which was consistent with the actual syndrome of the patient.

**Table 5:** Inference results of patient syndrome patterns.

Patient ID	Neighbors	Neighbors score
411	19	14.66
382	16	14.23
404	17	14.23
830	17	14.04
856	16	14.04
395	16	13.97
365	16	13.93
372	15	13.93
386	15	13.93
390	15	13.93
396	16	13.93
400	16	13.93
403	16	13.93
407	15	13.93
410	16	13.93
413	15	13.93
375	17	13.91
389	17	13.91
381	16	13.78
383	16	13.78

### Limitations

The proposed method considered the weight of common neighbors and the importance of different symptoms for different syndrome types, but this makes similarity calculation more complex, requiring more computing resources and time. Meanwhile, the calculation of edge weight values requires relatively rich and accurate feature data. If the data quality is not high or features are missing, it will affect the accuracy of similarity calculation. However, compared to large-scale knowledge graphs, our research has a smaller sample size and requires continuous data collection to enrich the knowledge base.

From the experimental results, our method achieved good results in the diagnosis of WFAI, LFBU, PFSI, and QDSS. However, some deficiencies existed in the differential diagnosis of QDSS and KED syndrome types, which could create confusion between the two. The analysis of 3 patients who were misclassified with KED instead of QDSS revealed common entities between them and the top 5 most similar patients among their neighbors (Textbox 1). The common entities between patient 1 (ID 415) and the top 5 most similar patients among their neighbors, who were all patients with QDSS but were misclassified with KED, are listed in Textbox 1. The common entities included worsening conditions when standing up, empty feeling in the ears, left side, worsening condition after physical exertion, hypertension, red tongue, anxiety, thin pulse, hearing loss, continuous symptoms, female sex, and dizziness. Similarly, patient 2 (ID 601) and the top 5 most similar patients among their neighbors shared common entities including worsening condition when standing up, empty feeling in the ears, left side, worsening condition after physical exertion, thin and white coating on the tongue, red tongue, anxiety, thin pulse, and continuous symptoms. Patient 3 (ID 423) and the top 5 most similar patients among their neighbors shared common entities including worsening condition after physical exertion, worsening condition at night, left side, use of headphones, exercise, pale tongue, thin coating on the tongue, tinnitus, middle to low frequency, and intermittent symptoms. By comparing the common entities between the patients and their top 5 most similar neighbors, we found that entities such as worsening condition after physical exertion and left side had higher scores in the differential diagnosis of the 2 syndrome types. However, ML algorithms were prone to confusion in the differential diagnosis because both QDSS and KED could be present in patients with these symptoms.

**Misclassified patient entity.**

**Patient 1 (ID 415)**

- Aggravation when standing up, ear emptiness, left side, aggravation after work, hypertension, tongue redness, anxiety, fine vein, hearing loss, duration, male, and dizziness.

**Patient 2 (ID 601)**

- Aggravation when standing up, ear emptiness, left side, aggravation after work, thin fur, white fur, tongue redness, anxiety, fine vein, and duration.

**Patient 3 (ID 423)**

- Aggravation after work, nighttime aggravation, left side, use headphones, exercise, tongue dullness, thin fur, cicada chirping, and interval.

### Conclusions

Tinnitus is a complex ear disease that poses challenging issues in clinical diagnosis due to the lack of specific indicators and the reliance on patient complaints. In this study, we constructed a medical knowledge graph based on EMRs and authoritative knowledge of patients with tinnitus and proposed an explainable tinnitus-assisted diagnosis model. The experimental results showed that our proposed method not only performed better in diagnostic performance with a diagnostic accuracy of over 98% for all syndromes but also offered better interpretability compared to general ML algorithms owing to the natural interpretability of the knowledge graph. Thus, the effectiveness of the proposed method was demonstrated to assist Chinese medicine doctors in diagnosing tinnitus during clinical practice.

## Abbreviations

AI: artificial intelligence
AUC: area under receiver operating characteristic curve
EMR: electronic medical record
KED: kidney essence deficiency
LFBU: liver fire bearing upward
ML: machine learning
PFSI: phlegm fire stagnation internally
QDSS: Qi deficiency of the spleen and stomach
TCM: traditional Chinese medicine
WFAI: wind fire attacking internally
